# Exploring the Role of Empathy in Understanding the Social-Cognitive Profile for Individuals Referred for Autism Spectrum Disorders Assessment in Adulthood

**DOI:** 10.1007/s10803-018-3693-8

**Published:** 2018-07-26

**Authors:** Victoria Russ, Hanna Kovshoff, Tony Brown, Patricia Abbott, Julie A. Hadwin

**Affiliations:** 1 Developmental Laboratory, Department of Psychology, Centre for Innovation in Mental Health, University of Southampton, Southampton, S017 1BJ UK; 2 Autism Diagnostic Research Centre, 9-19 Rose Road, Southampton, SO14 6TE UK

**Keywords:** Autism spectrum disorder, Adults, Neuropsychology, Social cognition, Diagnosis

## Abstract

This study explored the social-cognitive profile of 173 adults referred for an autism assessment. We considered key dimensional traits (autism, empathy and systemising) to understand social cognition in adults diagnosed with an autism spectrum condition compared with those who were referred for, but did not receive a diagnosis. There were no significant social cognitive differences between groups on measures of emotion recognition and social inference. Adults with a confirmed diagnosis, however, reported fewer empathising traits which were positively associated with social-cognitive understanding. Empathising partially mediated the relationship between diagnostic group and social-cognition. Lower empathising traits in individuals diagnosed in adulthood may be important in understanding challenges with social adaptability. The findings have implications for assessment and highlight the role of empathy in developing social understanding in autism.

## Introduction

Around 450,000 adults in the UK have a diagnosis of an Autism Spectrum Disorder (ASD) and a growing awareness of the disorder in adulthood has increased the demand for diagnostic services in adulthood (Howlin and Taylor 2015). Diagnosis in adulthood is reflected in recent changes to diagnostic criteria, notably, the removal of the criterion of a specific early age onset (American Psychiatric Association 2013). The increased demand for referrals and this diagnostic change present challenges for clinicians striving to deliver evidence-based assessments in a time-limited environment, and for researchers seeking to better understand the nature of adult ASD and associated difficulties (Russell et al. 2016; Scottish Autism Strategy 2011). In assessment settings, psychometric tools that have been standardised in the general population may provide meaningful information to clinicians who aim to develop a clear profile of the strengths and challenges that are faced by adults who are seeking a first-time ASD diagnosis (Bolte et al. 2011; Dell’Osso et al. 2016).

Changes in diagnostic criteria in ASD sit alongside a growing interest in dimensional approaches to diagnosis within development and psychopathology (Hudziak et al. 2007; Jalbrzikowski et al. 2017; Owen 2014). This shift from a categorical to a dimensional approach may represent a better fit with clinical practice, whereby diagnostic assessments tend to involve developing an individual profile of strengths and difficulties relating to social-communication and cognitive abilities in ASD (National Research Council 2001). Dimensional formulations of ASD have proposed that key traits of behaviour and ability exist in all individuals along a continuum (e.g. Skuse et al. 2004). In this conceptualisation, behaviour associated with ASD is proposed to sit at an ‘extreme’ end of a continuous distribution of multiple dimensions of autistic traits (Kamp-Becker et al. 2010). For example, the empathising–systemising (ES) framework was developed to capture the thinking style associated with ASD, while emphasising that these traits are also present to a greater or lesser extent in the typical population (Baron-Cohen 2009). This framework addresses the social and non-social features of ASD by highlighting variation in empathetic responding (i.e., differences or difficulties with responding emotionally to another person’s thoughts or feelings), alongside intact or superior skills in systemising (i.e. a drive to analyse, understand, predict, control and construct rule-based systems; see Baron-Cohen 2009).

Empathy reflects a degree of perspective taking to recognise another person’s emotion and to make relevant social inferences and appropriate emotional responses (Baez and Ibanez 2014). Empirical studies provide extensive support for a social-cognitive difference in ASD, that is most evident for individuals with increased severity of symptoms and developmental delay (review by Hadwin and Kovshoff 2015). For example, research has demonstrated difficulties in individuals with ASD in identifying others’ thoughts, (e.g. Wellman et al. 2001), recognising social faux pas (e.g. Baron-Cohen et al. 1999), and understanding non-literal expressions such as metaphors, sarcasm and lies (e.g. Kaland et al. 2002). While adults with average or above average intellectual functioning usually succeed in relatively simple social-cognitive tasks, research has demonstrated particular difficulties in advanced social-cognitive abilities. These can include, for example, understanding what one person thinks about another person’s thoughts, understanding non-literal expressions, and understanding the beliefs, intentions and meanings of indirect remarks or sarcasm (e.g. Mathersul et al. 2013).

Social-cognitive differences in individuals diagnosed with ASD are suggested to account for challenges linked to social adaptability observed in daily life, including difficulty initiating and sustaining friendships, making socially inappropriate comments, or misunderstanding social cues (e.g. Peterson et al. 2009). Further research has investigated the proposition that adults with ASD who do not have an intellectual impairment report or experience fewer social challenges and may learn and engage in social skills through logic and reasoning. Livingston and Happé (2017), for example, describe this process as “socially adapted behaviour” that may be “achieved via overt and conscious strategies, despite core socio-cognitive abilities, such as theory of mind, remaining impaired” (p. 733). In an earlier paper, Hofvander et al. (2009) proposed that the motivation to develop coping strategies to ‘mask’ autistic traits could stem from childhood negative experiences associated with not ‘fitting in’.

Early social experiences may lead to a type of “camouflaging” (Lai and Baron-Cohen 2015, p. 1013) that reflects observational learning of how to act in a social setting, using social rules and scripts, and that is underpinned by intellectual ability. The notion of camouflaging may be especially pronounced in adults with undetected ASD, who may reveal challenges in social adaptation via self-report, and where any difficulty is not immediately apparent from observation (Lai et al. 2011). This framework suggests that while core symptoms of ASD are present, they may be masked by learned strategies and/or may not become evident until social demands become increasingly complex and effortful. Researchers have further recognised that learned strategies create particular challenges in recognising ASD in adults, where difficulties in social-communication may go unnoticed without further exploration (Lai and Baron-Cohen 2015).

While a large body of research has aimed to understand the strengths and challenges of children and young people referred for an ASD assessment, research that has focused on adults who seek referral is sparse. An increasing number of adults being referred for or requesting an ASD diagnosis has led to calls for more understanding of this population, with the longer term goal of increasing quality of life and well-being (Howlin and Taylor 2015).

The present study aimed to extend current research to explore the social-cognitive profile of a population of individuals who were referred by their general practitioner to a specialist service for an ASD diagnostic assessment in adulthood and whose diagnosis, as part of this referral process, was confirmed or disconfirmed. The study investigated key social skills, including emotion recognition (recognising and labelling emotions from dynamic social scenes) and social inference (the ability to interpret conversational remarks meant literally or non-literally and to make judgments about the thoughts, intentions and feelings of others). Furthermore, it considered autism, empathy and systemising traits in diagnosed and non-diagnosed adults. It aimed to consider whether these dimensional traits differentiated group membership and were important in understanding any differences in the social-cognitive profiles in this population of adults. We utilised a measure of cognitive ability to establish that diagnostic groups were similarly matched. By building a profile of the relative strengths and challenges of adults diagnosed with ASD in adulthood, we aimed to inform the development of support and professional services that work to promote a better quality of life for this population.

## Method

### Participants

The study included a total of 192 adults aged between 18 and 75 years (*M*_*age*_ = 33.4 years, *SD* = 12.9; 76% male) who were referred by their general medical doctor/practitioner (GP) for an ASD assessment at a diagnostic service in the South of England between April 2008 and October 2014. All participants had provided written consent at the time of assessment for their anonymised data to be used for research purposes. Several adults were removed from the data as a result of (i) having an existing diagnosis of an intellectual disability and/or severe or enduring mental health problems (*N* = 6), (ii) where information regarding diagnostic outcome of assessment was missing (*N* = 8), and (iii) who were under 18 years old at the time of assessment (*N* = 6*)*.

Diagnosis was made using ICD-10 criteria (World Health Organization 1992) on the basis of a comprehensive assessment, undertaken by a team of trained professionals in accordance with National Institute of Clinical Excellence guidelines (NICE 2012). Several formal diagnostic tools were utilised with all individuals: the Adult Asperger Assessment (Baron-Cohen et al. 2005), the Autism Diagnostic Interview-Revised (Lord et al. 1994) and the Autism Diagnostic Observation Schedule (Lord et al. 1989). Following this assessment, 134 adults received a formal diagnosis of autism (*M*_age_ = 32.6, *SD* = 12.6, range 18–75 years; 75.4% male) and for 39 adults aged between 18 and 67 years (*M*_age_ = 36.4, *SD* = 13.4, range 18–67 years, 79.5% male) no formal diagnosis was given. There was no statistically significant difference in age between the ASD confirmed versus disconfirmed groups [*t* (172) = 1.68, *p* = .09].

We used data from the Wechsler Adult Intelligence Scale-IV (WAIS-IV) Digit Span to establish that the cognitive ability between diagnostic groups was similar (Wechsler 2008). It includes three tasks where individuals hear a sequence of numbers and are asked to recall the numbers in the same order (forward span), in reverse order (backward span), and then in ascending order (sequencing). It is suggested to measure working memory, as well as cognitive flexibility, rote memory and learning, attention, and encoding. Individuals can achieve one point for exact correct repetition of each trial of numbers. There are 16 trials (score 0–16); the scores from the three sub-tests are summed to produce a total subtest score which is transformed to an age-scaled score ranging from 1 to 19 (with mean = 10, SD = 3). Scores more than two standard deviations below the mean are considered to differ significantly from the general population. The Digit Span subtest has been shown to have excellent internal consistency and moderate to good test re-rest reliability (Wechsler 2008). In the current study there was no difference between groups on this subtest (*U* = 2350.00, *p* = .81; the median score for the diagnosed and undiagnosed groups was respectively 8 and 9; see Table 1 for descriptive statistics).

**Table 1:** Descriptive statistics for measures of cognitive ability, autism traits (autism traits, empathising and systematising) and social-cognitive measures (emotion understanding and social inference) for adults referred for an ASD diagnosis and where the diagnosis was confirmed or disconfirmed

Measure (cut-off)	ASD diagnosis confirmed				ASD diagnosis disconfirmed			
	Mean (± SD)	Range	N	Cut-off N (%)	Mean	Range	N	Cut-off N (%)
Cognitive ability								
Digit span (< 4)	8.73 (± 3.43)	3–18	130	4 (3)	8.24	3.57	37	5 (14)
Traits								
Autism* (> 32)	35.24 (± 7.49)	12–49	132	98 (74)	28.45 (± 9.52)	4–46	38	13 (34)
Empathy* (≤ 30)	19.33 (± 10.65)	1–62	132	104 (79)	24.53 (± 13.61)	4–65	38	28 (74)
Systemising	63.81 (± 27.20)	0–129	130	N/A	55.03 (± 24.44)	18–121	36	N/A
Social-cognitive measures								
Emotion evaluation (> 20)	22.06 (± 3.74)	11–28	103	83 (81)	22.21 (± 4.79)	9–27	29	21 (72)
Social inference (> 46)	48.95 (8.36)	2–65	120	88 (73)	48.70 (± 7.29)	34–60	30	22 (73)

Cognitive ability was measured using the Wechsler Adult Intelligence Scale-IV (WASI-IV;)—Digit Span (Wechsler 2008). The table shows scaled scores (mean = 10 and SD = 3)

*Indicates a significant group difference, *p* < .01

### Measures

#### ASD Traits

##### Autism

ASD traits were assessed using the Autism-Spectrum Quotient (AQ; Baron-Cohen et al. 2001). This self-report instrument includes 50 questions assessing five areas of functioning (i.e., social skills, attention switching, attention to detail, communication and imagination). Adults are asked how much they agree with each statement and score 1 for responses that reflect mild or strong agreement (score range 0–50 and higher scores indicate more autistic traits). The AQ is recognised as a screening tool for identifying undiagnosed cases of ASD (Allison et al. 2012; NICE 2012). In addition, it has been found to have good discriminant validity, where more than 80% of adults with a clinical diagnosis of ASD scored > 32 (/50) compared with 2% of a typical control group (Baron-Cohen et al. 2001). The mean AQ score in a non-clinical population is ~ 17 (/50; see review by Ruzich et al. 2015).

##### Empathising

The Empathy Quotient (EQ; Baron-Cohen and Wheelwright 2004) was used as a measure of adults’ cognitive and affective empathy. The EQ is a 40-item self-report questionnaire designed to measure how easily a person can detect and are affected by other people’s feelings. Participants indicate how much they agree with each item on a four-point Likert scale and empathic responses are scored either 1 or 2 (versus 0 for non-empathic responses; score range 0–80 and higher scores indicate increased empathy). Individuals diagnosed with ASD have been found to score significantly lower on the EQ (≤ 30) compared with typically developing populations (Baron-Cohen and Wheelwright 2004). The EQ has good psychometric properties (Lawrence et al. 2004; Allison et al. 2011).

##### Systemising

The Systemising Quotient-Revised (SQ-R; Wheelwright et al. 2006) was used to measure an individual’s preference for systemising. It comprises 75 questions that include observations of everyday events with a focus on the analysis of underpinning structures (e.g., “When I listen to a piece of music, I always notice the way it’s structured” or “When travelling by train, I often wonder exactly how the rail networks are coordinated.”). Individuals indicate how much they agree with each item on a four-point Likert scale from 0 (‘Strongly Disagree’) to 2 (‘Strongly Agree’). The range is 0–150, and higher scores indicate increased systemising. The SQ-R shows good psychometric properties (Groen et al. 2015). There is no published cut-off for this measurement.

#### Social Cognition

We used two sub-tests of The Awareness of Social Inference Test (TASIT; McDonald et al. 2002) to assess emotion recognition (The Emotion Evaluation Test; EE) and social inference (The Test of Social Inference—Minimal; SI). The EE test comprises 28 short videotaped vignettes of actors interacting in everyday situations and the participant is required to identify the actor’s emotion in each scene from a list of seven (happiness, sadness, anger, surprise, disgust, fear or neutral). Participants are given a score of 1 for a  correct response (score range 0–28). Normative data with typically developing adults found a median score of 25 (McDonald et al. 2003). Scores at or less than the 5th percentile (≤ 20; following McDonald et al. 2003) were used in the current paper as a cut-off to indicate low social cognitive ability.

The test of SI (minimal) is made up of 15 video vignettes, each lasting 15–60 seconds, that involve ambiguous conversational exchanges between two people, enacted to show either a sincere or a sarcastic response. For each vignette participants are asked what the actors in each scene are *thinking, doing, feeling*, and *saying* (score range 0–60, 1 point for each question across 15 vignettes). Normative data with typically developing individuals indicates a median score of 55 and we used scores at or less than the 5th percentile (≤ 46) as a cut-off for poor social inference skills (following McDonald et al. 2003). The TASIT has been shown to have good test–retest reliability (McDonald et al. 2006) and ecological validity (McDonald et al. 2004). It has also shown good validity as a measure of social cognition for adults diagnosed with ASD and whose IQ fall in the average range (Mathersul et al. 2013).

## Results

### Data Preparation and Analysis

Adults were grouped by diagnostic status; ASD confirmed or ASD disconfirmed. Because of the nature of the sample, all measures were not normally distributed. We therefore used non-parametric statistics to make basic comparisons between the diagnostic groups on self-reported measures of autistic traits, empathy, and systemising, as well as emotional and social inference skills^1^. In addition, we used published cut-off scores to consider the distribution of individuals who were above and below these in each group. Mediational analyses with bootstrapping (5000 samples; see MacKinnon et al. 2007) were conducted to further understand the relationship between key variables, with a view to identifying distinct social-cognitive profiles and associated pathways (via autism, empathising and systemising traits) between the diagnosis confirmed and diagnosis disconfirmed groups.

### Group Differences in ASD Traits

Table 1 shows the mean scores for each group and the percentage of participants whose score was within the clinical range according to cut-off points for autism and empathy traits. Comparing groups, adults with a confirmed diagnosis of ASD self-reported more autistic traits (Mann–Whitney *U* = 1390.00, *p* < .001; Mdns for the diagnosed and non-diagnosed groups were 36 and 28 respectively). There was also a significant group difference for empathy with the diagnosed group reporting fewer empathic traits (Mann–Whitney *U* = 1943.50, *p* = .035, and respective medians = 17 and 22). The results also showed a marginal non-significant effect for systemising traits, with the diagnosed group reporting more traits (Mann–Whitney *U* = 1898.00, *p* = .083, and respective medians = 63 and 48).

Considering the distribution of individuals across cut-off points for all measures, and between groups, analysis showed that an increased proportion of adults in the diagnosed group reported scores above the cut-off (versus those below the cut-off) for autistic traits and compared with those in the non-diagnosed group ($\chi$^2^ = 20.87, *p* < .001). No other comparisons were significant (see Table 1).

### Group Differences in Social-Cognitive Functioning

There were no significant group differences on measures of emotion evaluation (EE) or social inference (SI; in both cases *p* > .4). Mdn scores for the EE and SI tests for the diagnosed and non-diagnosed groups were respectively 23 and 24, 50 and 50.

### Associations Between Variables

A non-parametric correlational analysis was conducted to explore the associations between variables, including diagnostic group, cognitive ability, dimensional traits, and indices of social-cognitive functioning. Significant positive correlations were found between diagnostic group (coded as 2 = diagnosis confirmed and 1 = diagnosis disconfirmed) with ASD and self-reported autism traits (see Table 2). Empathic traits were significantly negatively associated with diagnostic group and positively linked to measures of emotion recognition. We therefore considered whether empathising was an important prerequisite ability accounting for variation in emotion understanding between our groups.

**Table 2:** Inter-correlations between diagnostic group (adults referred for an ASD diagnosis and where the diagnosis was confirmed or disconfirmed) with ASD traits (autism traits, empathising and systematising) and social-cognition indices

	1	2	3	4	5	6	7
1. Diagnostic group	–	− .003	.32**	− .16*	.14	− .03	− .01
Cognitive ability							
2. Digit span		–	− .12	.23	.21	.30	.32^#^
Traits							
3. ASD traits			–	− .70**	.46**	− .11	− .10
4. Empathising ability				–	− .30**	.18*	.12
5. Systemising tendency					–	.04	− .03
Social-cognitive measures							
6. Emotion evaluation						–	.57**
7. Social inference							–

Statistics indicate *Spearmans rho*, Ns vary between measures (see Table 1), ^#^*p* < .1,, *p < .05,, **p < .001

### Understanding the Relationship between Empathising Traits and Emotion Understanding

**Fig. 1:**
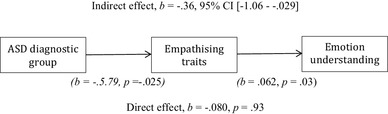
Mediation analyses exploring the relationship between diagnostic group (ASD confirmed and ASD disconfirmed) and emotion understanding via mediated self-reported empathising traits. Indirect effect, *b* = − .36, 95% CI [− 1.06 to − .029] *(b* = − .*5.79, p* = − .025*) (b* = .062, *p* = .03). Direct effect, *b* = − .080, *p* = .93

We tested a mediational model to consider whether there were indirect links between diagnostic groups with emotional understanding via empathising traits. Mediational analyses were conducted using the PROCESS method (Hayes 2013). Consistent with the analysis above, there was no direct association between groups with social understanding. The results did, however, show a significant indirect effect of diagnostic group on emotion understanding ability via empathy traits (*b* = − .36, 95% CI [− 1.06 to − .029]), indicating that empathising partially mediated the relationship between diagnostic group and emotion understanding (see Fig. 1).

## Discussion

This study explored social cognition of adults referred to a diagnostic service for an ASD assessment in adulthood. We considered the social-cognitive profile between adults whose ASD diagnosis was confirmed versus those where the diagnosis was not confirmed. In addition, we explored whether individual differences in empathy, as well as autism and systemising traits, were important in explaining emotion understanding and social inference in the two diagnostic groups. The results showed that adults in the ASD diagnosis confirmed group reported more autism traits and fewer empathic skills, compared to adults whose diagnosis was not confirmed. There was a marginal (but not statistically significant) group difference in systemising traits, with the ASD diagnosed group endorsing a greater number. Moreover, fewer self-reported empathic skills were found to be important in mediating social-cognitive differences between diagnostic groups. The results suggest that underpinning empathy differences between diagnostic groups was important in understanding individual differences in emotion understanding.

Consistent with the empathising–systemising theory (Baron-Cohen 2009), the current findings indicated that adults referred for assessment and who received a diagnosis of ASD presented with relative strengths in systemising, alongside poorer empathic skills. Baron-Cohen and Wheelwright (2004) argued that empathy is a core skill that facilitates effective social interaction, and that underpins the development of social relationships and prosocial behaviour. Consequently, lower empathic ability may reflect less social adaptability (e.g., relationship or friendship difficulties/breakdown), and may be a key factor in understanding reasons for referral in adulthood (Sasson et al. 2013). Geurts and Jansen (2011) identified that social difficulties were one of the most common reasons for referral to an ASD assessment service, and the current findings suggest that these challenges may arise from difficulties in empathising associated with social interaction. This finding is consistent with recent work in the general population, where social difficulties mediated the relationship between autistic traits and income level (Skylark and Baron-Cohen 2017).

Some researchers have suggested that a diagnosis of ASD in adulthood is associated with fewer autistic traits (e.g. Seltzer et al. 2004), in addition to individuals showing average or above average intellectual functioning (e.g. Aggarwal and Angus 2015). The current study showed that most adults across the sample showed cognitive abilities within a typical range. In addition, cognitive ability was not different between diagnostic groups, but the number of adults endorsing autism traits was greater in the ASD diagnosed group. Clinicians familiar with a particular profile of strengths and deficits in social-cognition, typically seen in ASD, may miss this different presentation in adults seeking first-time diagnosis. NICE guidelines (2012) endorse the use of self-report measures of autistic traits and empathising ability to aid the complex task of assessment and diagnosis of adults. Consistently, researchers have also supported the use of dimensional measures of cognitive and social functioning in addition to an ASD diagnosis (e.g., Volkmar et al. 2009). The current study supports the exploration of traits using the AQ and EQ screening measures as part of a comprehensive assessment in adults who present for an ASD diagnosis in adulthood.

Although an ASD diagnosis was not directly associated with emotion understanding, lower empathic skills were found to help explain the profile of social-cognitive difficulties for individuals referred for an ASD assessment in adulthood. This finding highlights the important role of understanding dimensional ASD traits, rather than a diagnostic category alone, in interpreting social functioning. An implication is that clinicians should be aware that measures assessing social-cognition may not be sensitive enough to detect difficulties in functioning for adults seeking first-time diagnosis. The findings fit with the proposition of compensation in adult ASD and where adults living with ASD can show intact cognitive ability and good social reasoning skills, but where underpinning psychological constructs are less evident (see Lai and Baron-Cohen 2015; Livingstone and Happé 2017). Consistently, adults diagnosed with ASD in adulthood (versus childhood) have reported that their higher cognitive ability enabled them to use logical reasoning to overcome their difficulties in social functioning (Lovett 2005). This camouflaging of autistic behaviours causes challenges for clinicians attempting to explore the social presentation typically associated with ASD (Bastiaansen et al. 2011), not withstanding the difficulties autistic individuals themselves report experiencing as a function of effortfully masking autistic traits to ‘fit in’ (Hull et al. 2017). Given the potential increased risk for stress, negative impact on self-esteem and exhaustion for adults using these strategies, camouflaging should be neither expected nor encouraged as an intervention (Lai et al. 2011; Hull et al. 2017).

The findings of the current study indicate that adults who receive a diagnosis of ASD in adulthood may welcome an opportunity to develop social skills and where the focus is on empathic processing. A recent review of empathy focused interventions for health professionals indicated that it is possible to enhance empathic skills in adults to increase their social-cognitive understanding via empathic processes utilising role play, video and discussion (Kiosses et al. 2016). Future research should aim to understand if opportunities to explore the role of empathy in the context of social-cognition would be acceptable or beneficial for adults referred for a diagnosis of ASD. In addition, further studies are required to understand the extent to which empathic processes are sensitive to change via intervention programmes.

Alongside possible opportunities to enhance the skills of the person with autism, more emphasis has recently been placed on creating a better ‘person-environment’ fit (Lai and Baron-Cohen 2015). In this case, the focus is on how social contexts react to autistic people. This approach reflects the ‘double empathy problem’, which acknowledges the challenges individuals with autism face in processing and understanding other people, but also highlights that non-autistic individuals need to work harder to understand autistic individuals (Milton 2018). This framework suggests that there is a mutual and reciprocal misunderstanding of both parties, due to experiencing the world in very different ways. Specifically, Milton (2018) proposes that targets for intervention and further research should focus on empowering individuals with autism, to fostering an understanding and appreciation of their world view, and to bridge the ‘double-empathy gap’ by developing shared interactional expertise.

There are several limitations to this study. Studies to compare diagnosed and nondiagnosed adults who have been referred for assessment in adulthood are sparse; therefore, the findings require independent replication. Given the substantial heterogeneity within ASD, and the focus of the present study on adults with no recorded intellectual disability, one caveat is whether the results from this subgroup of adults will generalise to individuals diagnosed with ASD more broadly. A further limitation of the present study design was that historical information was not obtained for the referred sample about previous diagnoses, referrals or assessments.

Despite these limitations, the study has strength in the large sample size, wide age-range of adults, use of a clinical population, and the clinical relevance of the findings. This novel study addresses the call for real-world research and contributes to furthering our dimensional understanding of the strengths and challenges for adults seeking first-time ASD diagnosis.

## Footnotes

1 Across *N* = 173 adults the number who completed each measure varied. For the autism trait questionnaires *N* = 167 and *N* = 170. For the TASIT EE and SI tests the numbers were *N* = 132 (EE) and *N* = 150 (SI).
